# Modelling infectious diseases with herd immunity in a randomly mixed population

**DOI:** 10.1038/s41598-021-00013-2

**Published:** 2021-10-18

**Authors:** Kian Boon Law, Kalaiarasu M. Peariasamy, Hishamshah Mohd Ibrahim, Noor Hisham Abdullah

**Affiliations:** 1 Institute for Clinical Research, National Institutes of Health, Ministry of Health Malaysia, Setia Alam, Malaysia; 2 The Office of Director General, Ministry of Health Malaysia, Putrajaya, Malaysia

**Keywords:** Epidemiology, Computational models, Infectious diseases

## Abstract

The conventional susceptible-infectious-recovered (SIR) model tends to magnify the transmission dynamics of infectious diseases, and thus the estimated total infections and immunized population may be higher than the threshold required for infection control and eradication. The study developed a new SIR framework that allows the transmission rate of infectious diseases to decline along with the reduced risk of contact infection to overcome the limitations of the conventional SIR model. Two new SIR models were formulated to mimic the declining transmission rate of infectious diseases at different stages of transmission. Model A utilized the declining transmission rate along with the reduced risk of contact infection following infection, while Model B incorporated the declining transmission rate following recovery. Both new models and the conventional SIR model were then used to simulate an infectious disease with a basic reproduction number (r_0_) of 3.0 and a herd immunity threshold (HIT) of 0.667 with and without vaccination. Outcomes of simulations were assessed at the time when the total immunized population reached the level predicted by the HIT, and at the end of simulations. Further, all three models were used to simulate the transmission dynamics of seasonal influenza in the United States and disease burdens were projected and compared with estimates from the Centers for Disease Control and Prevention. For the simulated infectious disease, in the initial phase of the outbreak, all three models performed expectedly when the sizes of infectious and recovered populations were relatively small. As the infectious population increased, the conventional SIR model appeared to overestimate the infections even when the HIT was achieved in all scenarios with and without vaccination. For the same scenario, Model A appeared to attain the level predicted by the HIT and in comparison, Model B projected the infectious disease to be controlled at the level predicted by the HIT only at high vaccination rates. For infectious diseases with high r_0_, and at low vaccination rates, the level at which the infectious disease was controlled cannot be accurately predicted by the current theorem. Transmission dynamics of infectious diseases with herd immunity can be accurately modelled by allowing the transmission rate of infectious diseases to decline along with the reduction of contact infection risk after recovery or vaccination. Model B provides a credible framework for modelling infectious diseases with herd immunity in a randomly mixed population.

## Introduction

Herd or population immunity refers to the indirect protection among susceptible individuals when most people in a population have become immune to an infectious disease either by vaccine immunity or natural immunity. The concept of herd immunity became a fixture of epidemiology in the 1930s, and later popularized in 1950s and 1960s for public health policy decisions for the introduction of new vaccines to eradicate infectious diseases^1^. Herd immunity takes effect when the transmission rate of infectious diseases declines along with the reduced risk of infection due to the presence or proximity of immune individuals in a randomly mixed population^2^. Although herd immunity is observed at population level in diseases, such as measles, mumps, rubella, pertussis, chickenpox and polio, the mathematical predictions have not been consistent through modelling.

The susceptible-infectious-recovered (SIR) mathematical models are widely used to simulate the transmission pattern of infectious diseases. These models use a flexible compartmental framework with robust assumptions for a wide range of applications^3–6^. The SIR compartmental framework simplifies the transmission dynamics of infectious diseases by classifying individuals based on their epidemiological status and ability to host and transmit pathogens^7^. The SIR model also assumes that complete immunity can be acquired through infection, hence encompassing the epidemiological notion of natural herd immunity^8,9^.

The concept of herd immunity assumes that an infectious disease can be controlled or eradicated when the total immunized population reaches the herd immunity threshold (HIT) level. Beyond the HIT, the transmission of the infectious disease becomes unsustainable as one infected individual would generate less than one secondary case on average^10^. The HIT can be calculated from the basic reproduction number (r_0_) of an infectious disease to guide the vaccination strategy for an endemic or pandemic^2,11^. For instance, in COVID-19 pandemic, the vaccination strategy should cover a minimum of 50.0% to 66.7% of the population based on the assumption that the r_0_ of 2.0 to 3.0 for the novel coronavirus^12–15^.

One of the shortcomings of the conventional SIR model is that it tends to magnify the transmission dynamics of infectious diseases. For instance, for an infectious disease with r_0_ of 3.0, the SIR model estimates that up to 94.0% of a population shall become infected. The estimated level is beyond the expected HIT, even with the presence of natural herd immunity, thereby overdrawing public health planning and preparedness for the COVID-19 pandemic^16–19^. This study aims to investigate and overcome the aforesaid limitation of the SIR model in modelling infectious diseases with herd immunity in a randomly mixed population. We propose a key modification to the conventional SIR model that allows the transmission rate of infectious disease to decline along with the reduced risk of contact infection in line with the principle of herd immunity.

## Methods

### Conventional SIR model

**Figure 1:**
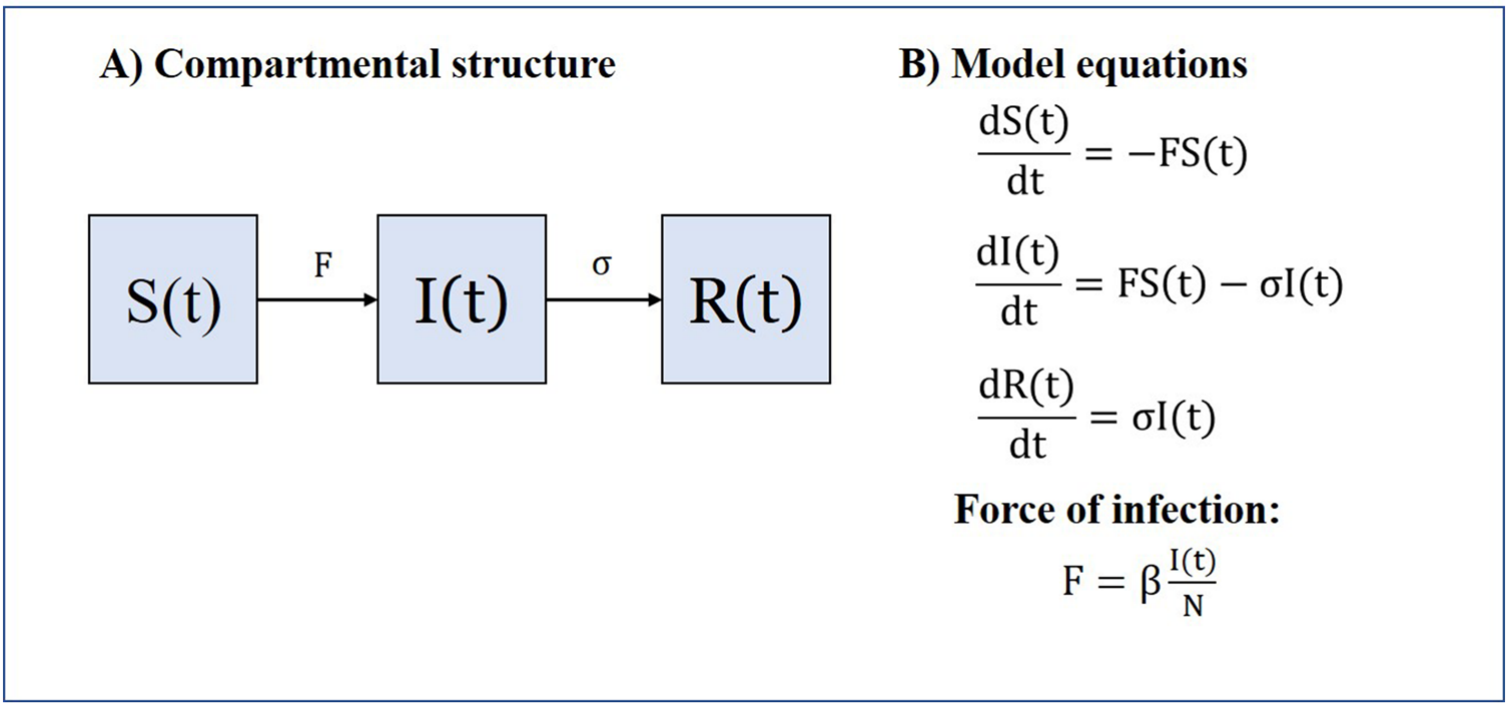
The compartmental structure and model equations of Kermack & Mckendrick’s SIR model.

Kermack & McKendrick postulated the first SIR model for infectious diseases in 1927 before vaccines became popular in the 1950s for the control and eradication of infectious diseases^9^. Later, the SIR model formed the basis of most infectious disease models whereby the model divides a homogenous population (N) into three basic states or compartments: susceptible denoted by S(t), infectious denoted by I(t), and recovered or removed denoted by R(t), and assumes infectious diseases spread from affected to unaffected individuals through contact infection (Fig. 1). Susceptible individuals have equal risk of being infected. Infectious are infected individuals who have developed infectivity and can transmit pathogens to susceptible individuals. Recovered or removed are individuals who have recovered from infection and protected from reinfection by natural immunity. In brief, the conventional SIR model describes the transmission dynamics of infectious diseases with natural immunity through infection. The SIR model can be described mathematically by a set of ordinary differential equations (ODEs) as shown in Fig. 1.

According to the ODEs as presented in Fig. 1, the total rate of individuals moving from compartment S(t) to I(t) due to contact infection is determined by S(t) and the force of infection, F, which consists of the product of the constant infection rate, β, and the proportion of infectious individuals, $\frac{\mathrm{I}(\mathrm{t})}{\mathrm{N}}$ at time t. And, the total rate of individuals moving from compartment I(t) and R(t) is determined by I(t) and the recovery rate, σ denoted by the reciprocal of infection duration. Therefore, the conventional SIR model often simulates the I(t) to increase at the initial phase of an outbreak and subsequently diminishes due to the exhausted stock of S(t).

Without vital dynamics, the total population size is given by,

1 $${\text{N }} = {\text{ S}}\left( {\text{t}} \right) \, + {\text{ I}}\left( {\text{t}} \right) \, + {\text{ R}}\left( {\text{t}} \right).$$

Equation (1) can be converted into prevalence or proportion by dividing each notation with the total population size, N:

2 $$1=\frac{\mathrm{S}(\mathrm{t})}{\mathrm{N}}+\frac{\mathrm{I}(\mathrm{t})}{\mathrm{N}}+\frac{\mathrm{R}(\mathrm{t})}{\mathrm{N}}.$$

According to Eq. (2), I(t) and R(t) are often very small as compared with N at the beginning of the transmission, therefore, $\frac{\mathrm{I}(\mathrm{t})}{\mathrm{N}}\approx 0$, $\frac{\mathrm{R}(\mathrm{t})}{\mathrm{N}}\approx 0$, and $\frac{\mathrm{S}(\mathrm{t})}{\mathrm{N}}\approx 1$. At the end of transmission, the I(t) would become very small again, therefore, $1-\frac{\mathrm{R}(\mathrm{t})}{\mathrm{N}}\approx \frac{\mathrm{S}(\mathrm{t})}{\mathrm{N}}$. To simulate infectious diseases with herd immunity, $\frac{\mathrm{S}(\mathrm{t})}{\mathrm{N}}$ or $1-\frac{\mathrm{R}(\mathrm{t})}{\mathrm{N}}$ can be incorporated into the force of infection, F to model the reduced risk of contact infection at different stages of infection.

By incorporating $\frac{\mathrm{S}(\mathrm{t})}{\mathrm{N}}$ into the F, we assume herd immunity takes effect to reduce the transmission rate of infectious diseases following infection. By incorporating $1-\frac{\mathrm{R}(\mathrm{t})}{\mathrm{N}}$ into the F, we assume herd immunity takes effect to reduce the transmission rate of infectious diseases following recovery.

### New SIR models

The Law of Mass Action describes the rate of chemical reactions being proportional to the concentration of reactants^20^. Based on this principle, contact infection can be regarded as an interactive event between susceptible and infectious individuals in a randomly mixed environment, with the rate being proportional to both $\frac{\mathrm{S}(\mathrm{t})}{\mathrm{N}}$ and $\frac{\mathrm{I}(\mathrm{t})}{\mathrm{N}}$. The product of $\frac{\mathrm{S}(\mathrm{t})}{\mathrm{N}}$ and $\frac{\mathrm{I}(\mathrm{t})}{\mathrm{N}}$ denotes the combined risk of contact infection, hence, two new models can be formulated.

#### Model A

The total transmission rate of infectious diseases in a randomly mixed population depends on the S(t) and the new force of infection, F_A_, which is the product of β, $\frac{\mathrm{I}(\mathrm{t})}{\mathrm{N}}$ and $\frac{\mathrm{S}(\mathrm{t})}{\mathrm{N}}$ as follow:

3 $${\mathrm{F}}_{\mathrm{A}}=\upbeta \frac{\mathrm{I}(\mathrm{t})\mathrm{S}(\mathrm{t})}{{\mathrm{N}}^{2}}.$$

**Figure 2:**
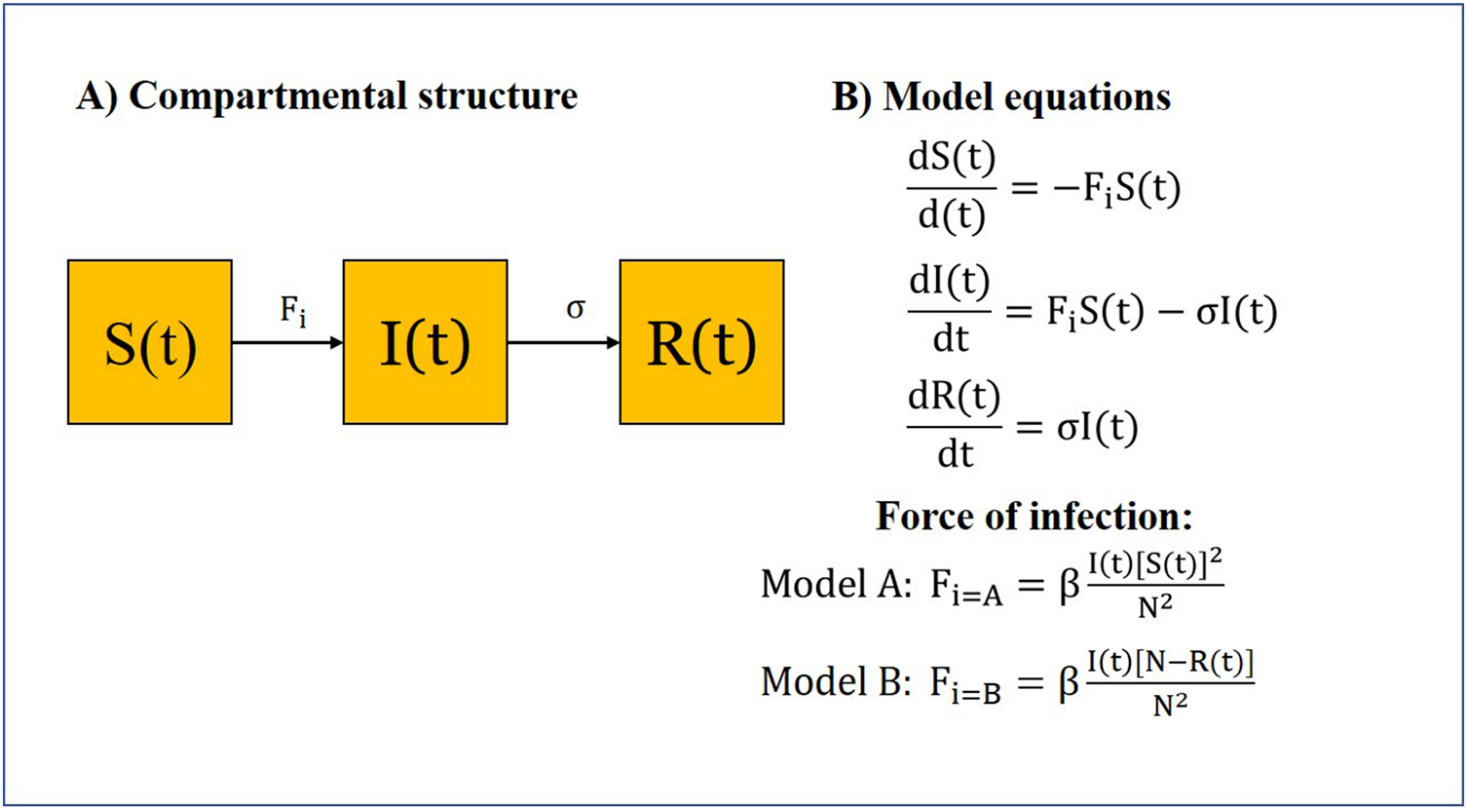
The compartmental structure and model equations of the newly developed Model A and Model B.

In Model A, the risk of contact infection is determined by both $\frac{\mathrm{S}(\mathrm{t})}{\mathrm{N}}$ and $\frac{\mathrm{I}(\mathrm{t})}{\mathrm{N}}$. Therefore, the transmission rate would decline along with the reduced risk of contact infection when infected individuals move from compartment S(t) to I(t). The compartmental structure and model equations of Model A can be found in Fig. 2.

#### Model B

The total transmission rate of infectious diseases in a randomly mixed population depends on the S(t) and the new force of infection, F_B_, which is the product of β, $\frac{\mathrm{I}(\mathrm{t})}{\mathrm{N}}$ and $1-\frac{\mathrm{R}(\mathrm{t})}{\mathrm{N}}$ as follow:

4 $${\mathrm{F}}_{\mathrm{B}}=\upbeta \frac{\mathrm{I}(\mathrm{t})\left[\mathrm{N}-\mathrm{R}(\mathrm{t})\right]}{{\mathrm{N}}^{2}}.$$

In Model B, the risk of contact infection is determined by both $1-\frac{\mathrm{R}(\mathrm{t})}{\mathrm{N}}$ or $\frac{\mathrm{N}-\mathrm{R}(\mathrm{t})}{\mathrm{N}}$ and $\frac{\mathrm{I}(\mathrm{t})}{\mathrm{N}}$. Therefore, the transmission rate would decline along with the reduced risk of contact infection when individuals move from compartment I(t) to R(t) after recovery. The $1-\frac{\mathrm{R}(\mathrm{t})}{\mathrm{N}}$ denotes the inverse proportion of recovered individuals that captures the reduced risk of contact infection following recovery. The compartmental structure and model equations of Model B can be found in Fig. 2.

Both Model A and Model B retain the SIR compartmental structure, except for the force of infection (Fig. 2). With the modification, both models can be used to simulate the transmission dynamics of infectious diseases with natural herd immunity in a randomly mixed population.

### The basic reproduction number, r_0_

The equations of I(t) from the conventional SIR model, Model A and Model B can be rearranged as follows:

5 $$\frac{\mathrm{dI}(\mathrm{t})}{\mathrm{dt}}=\left[\upbeta \frac{\mathrm{S}(\mathrm{t})}{\mathrm{N}}-\upsigma \right]\mathrm{I}(\mathrm{t}).$$

6 $$\frac{\mathrm{dI}(\mathrm{t})}{\mathrm{dt}}=\left[\upbeta {\left(\frac{\mathrm{S}(\mathrm{t})}{\mathrm{N}}\right)}^{2}-\upsigma \right]\mathrm{I}(\mathrm{t}).$$

7 $$\frac{\mathrm{dI}(\mathrm{t})}{\mathrm{dt}}=\left[\upbeta \left(\frac{\mathrm{S}(\mathrm{t})\left[\mathrm{N}-\mathrm{R}(\mathrm{t})\right]}{{\mathrm{N}}^{2}}\right)-\upsigma \right]\mathrm{I}(\mathrm{t}).$$

At the beginning of the transmission, when $\frac{\mathrm{S}(\mathrm{t})}{\mathrm{N}}\approx 1$ and $\frac{\mathrm{R}(\mathrm{t})}{\mathrm{N}}\approx 0$, we would obtain the exact equation for all three models as follow,

8 $$\frac{\mathrm{dI}(\mathrm{t})}{\mathrm{dt}}=\left(\upbeta -\upsigma \right)\mathrm{I}(\mathrm{t}).$$

The integral of Eq. (8) is an exponential function as follow,

9 $$\mathrm{I}(\mathrm{t})={\mathrm{I}}_{0}{\mathrm{e}}^{(\upbeta -\upsigma )\mathrm{t}}.$$

Equation (9) shows a crucial condition that determines the widespread of infectious diseases in a population. The transmission of infectious diseases can be sustained if β > σ or $\frac{\upbeta }{\upsigma }>1$. The ratio between β and σ denotes the basic reproduction number (r_0_) of infectious diseases. The r_0_ can also be defined as the number of secondary cases caused by a single primary case in a wholly susceptible population^21,22^. The r_0_ can be used to derive the herd immunity threshold (HIT) according to a simple theorem proposed by Dietz (1975)^23^.

10 $$\mathrm{HIT}=1-\frac{1}{{\mathrm{r}}_{0}}.$$

The HIT can also be defined as the level that the transmission of infectious diseases becomes unsustainable as one infected person generates less than one secondary case on average in a population^10^. Often, the HIT can predict total infections achieved at the end of transmission without vaccination. If vaccination is used to control the spread of infectious diseases, the HIT indicates the share of a population that needs to be vaccinated.

### Vaccine models

**Figure 3:**
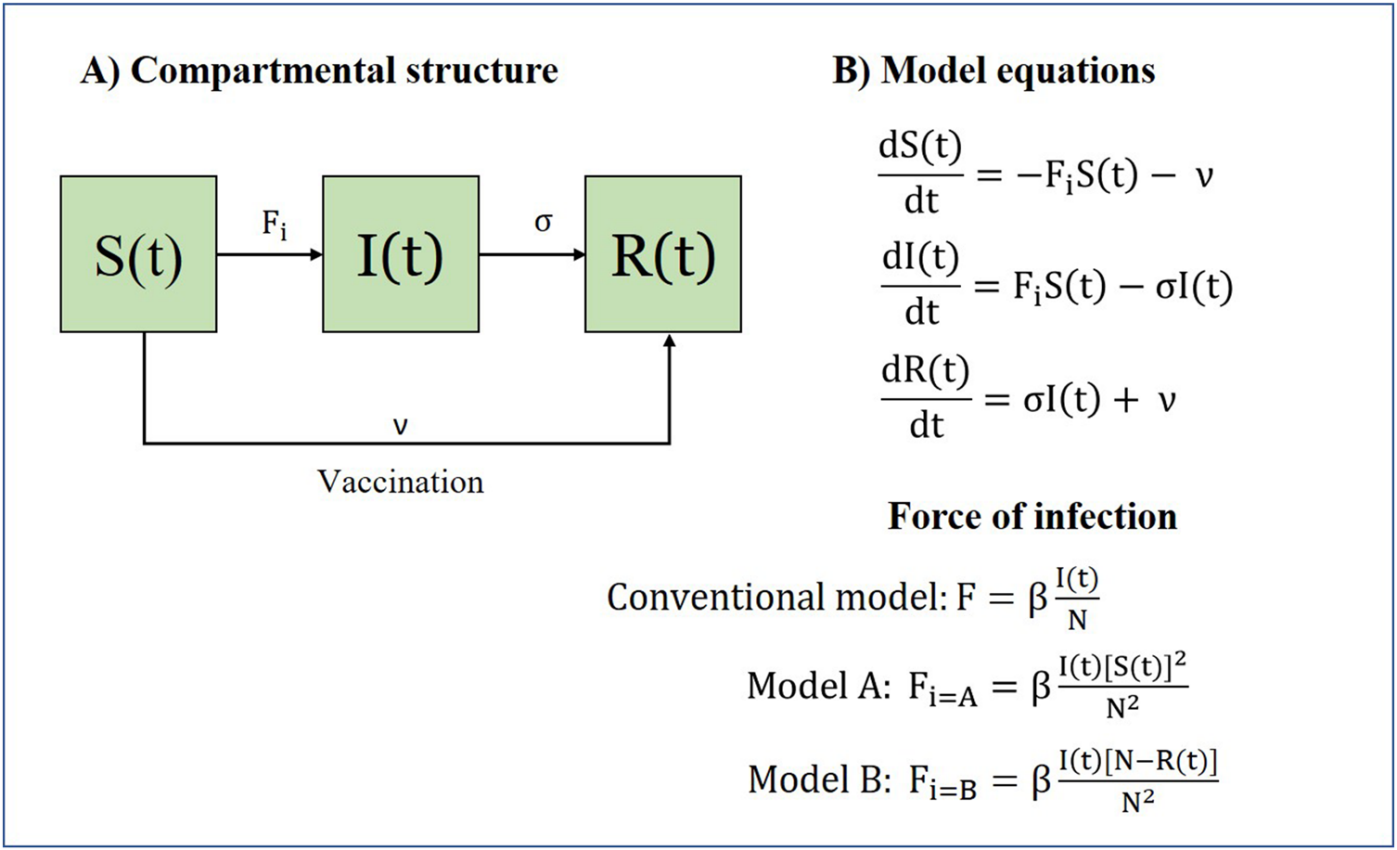
The compartmental structure and model equations of vaccine models modified using the conventional SIR model, Model A and Model B.

Unlike natural immunity, vaccine introduces immunity into individuals without developing infectivity, therefore protecting a significant portion of population from more infections. A simple vaccine model can be created using the conventional SIR model, Model A and Model B by allowing vaccinated individuals to move from compartment S(t) straight to R(t) at a constant vaccination rate denoted by ν (Fig. 3). The magnitude of ν depends on factors such as the availability of vaccines and resources for vaccination, not the size of S(t). Here, we assume vaccinated individuals would develop complete immunity as those who have been infected. Therefore, the compartment R(t) would consist of the total immunized population with natural and vaccine-induced immunity. Herd immunity is considered achieved when the total immunized population reaches the HIT.

Table 1 shows the total transmission and recovery rates of the conventional SIR model, Model A and Model B. By assigning the same value to β, σ and ν, all three models can be used to simulate the transmission dynamics of an infectious disease with herd immunity either through infection or vaccination in a randomly mixed population.

**Table 1:** Breakdown of total transmission rate and recovery rate of the conventional SIR model, Model A and Model B.

Models	Components of total transmission rate			Total transmission rate from S(t) to I(t)	Components of total recovery rate		Total recovery rate from I(t) to R(t)	Vaccination rate
	Force of infection		Number of susceptible					
	Infection rate	Risk of contact infection			Recovery rate	Number of Infectious		
Conventional SIR model	β	$\frac{\mathrm{I}(\mathrm{t})}{\mathrm{N}}$	S(t)	$\upbeta \frac{\mathrm{I}(\mathrm{t})\mathrm{S}(\mathrm{t})}{\mathrm{N}}$	σ	I(t)	$\mathrm{\upsigma I}(\mathrm{t})$	ν
Model A	β	$\frac{\mathrm{I}(\mathrm{t})\mathrm{S}(\mathrm{t})}{{\mathrm{N}}^{2}}$	S(t)	$\upbeta \frac{\mathrm{I}(\mathrm{t}){\left[\mathrm{S}(\mathrm{t})\right]}^{2}}{{\mathrm{N}}^{2}}$	σ	I(t)	$\mathrm{\upsigma I}(\mathrm{t})$	ν
Model B	β	$\frac{\mathrm{I}\left(\mathrm{t}\right)[\mathrm{N}-\mathrm{R}(\mathrm{t})]}{{\mathrm{N}}^{2}}$	S(t)	$\upbeta \frac{\mathrm{I}\left(\mathrm{t}\right)\mathrm{S}\left(\mathrm{t}\right)[\mathrm{N}-\mathrm{R}(\mathrm{t})]}{{\mathrm{N}}^{2}}$	σ	I(t)	$\mathrm{\upsigma I}(\mathrm{t})$	ν

### Simulations and sensitivity analyses

The ODEs of all three models can be solved via numerical integration. First, we simulated all three models under the exact and arbitrary conditions with parameter values as presented in Table 2. These parameter values allowed all the models to project the transmission dynamics of the same infectious disease in a homogenous population. We assumed herd immunity can be achieved either through infection or vaccination. Herd immunity was considered achieved when the total immunized population reached the HIT.

**Table 2:** Parameter values used in simulations and sensitivity analyses.

Parameters	Values
Infection rate, β	0.3 0.11 to 1.00 (sensitivity analysis)
Recovery rate, σ	0.1
Infection duration	10
Basic reproduction number, r_0_	3.0 1.1 to 10.0 (sensitivity analysis)
Herd immunity threshold, HIT	0.667 (66.7%)
Vaccination rate, ν	1.0% population per unit t 0.5% population per unit t 0.1% population per unit t
Population size, N	1,000,000
Initial value for I(t)	1
Initial value for S(t)	N − I(t)
Initial value for R(t)	0
Initial value for total infections	1

The transmission dynamics of the infectious disease with natural herd immunity were simulated using models as presented in Figs. 1 and 2. We expected the infectious disease to subside when the total immunized population or R(t) reached the level predicted by the HIT. We evaluated the size of each compartment at the time when the HIT was reached, and at the end of simulation (t = 200). The transmission dynamics of the infectious disease with herd immunity through vaccination and infection were simulated using vaccine models as presented in Fig. 3. The total immunized population would consist of those who had developed natural immunity and vaccine immunity. At high vaccination rates, the total immunized population was largely contributed by those acquiring vaccine immunity. At low vaccination rates, the total immunized population was largely contributed by those acquiring natural immunity. We simulated all vaccine models at three vaccination rates, as stated in Table 2. At each vaccination rate, we evaluated the size of each compartment at the time when the HIT was reached, and at the end of simulations (t = 500 for ν = 1.0%, t = 300 for ν = 0.5%, and t = 200 for ν = 0.1%). The vaccination rate was set to zero after the HIT was reached until the end of simulations. In sensitivity analyses, we evaluated total infections generated by all three models at the end of simulation across r_0_ values from 1.1 to 10.0 without vaccination.

In an effort to validate the new model, the conventional SIR model, Model A and Model B were used to simulate the transmission dynamics of seasonal influenza in the United States. The disease burden of symptomatic illness as projected by the models at equilibrium state were compared with estimates reported by the Centers for Disease Control and Prevention (CDC) from 2010 to 2019^24^. We assumed complete immunity was acquired after recovery. Subsequently, we applied a basic reproduction number (r_0_) of 1.3 for seasonal influenza^25^, contagiousness period of 5 days^26^, annual vaccination coverage of 50% population^27^, vaccine effectiveness of 60%^28^, and full vaccine immunity developed after 14 days and lasted for at least 6–12 months^29^. The simulations were conducted for 15 years, starting from a total population size of 295.5 million, mirroring the US population size in 2005 with an approximate net growth rate of 0.5% annually^30^. Equilibrium states were expected to be achieved after 5 years (t = 1826) and the disease burden projected by the three models from 6 to 15 years (t = 1826 to 5475) were compared with estimates reported by the CDC from 2010 to 2019. Numerical integrations and simulations were performed in R version 4.1.0 with “deSolve” package^31,32^. Graphics were prepared in Microsoft Excel 2019 (version number: 16051.14326.20348.0). All scripts used to perform simulations and datasets can be downloaded from the GitHub repository of the research at https://github.com/william81/newSIR.herd-immunity.model.script.

### Ethics requirement

The study was registered with the National Medical Research Register. No ethics approval was required.

## Results

### Transmission dynamics of infectious diseases with natural herd immunity

**Figure 4:**
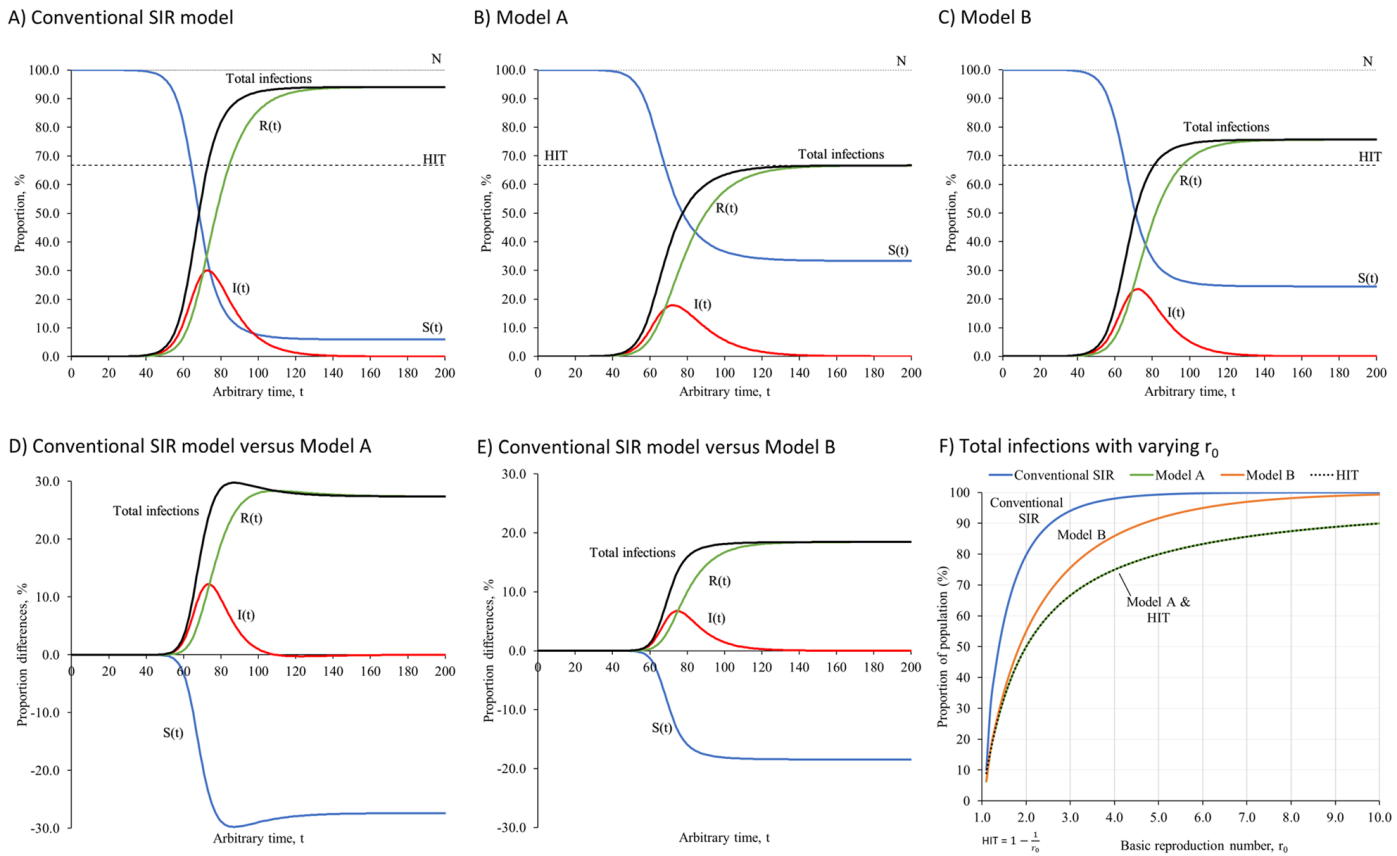
The transmission dynamics of infectious diseases with natural herd immunity. **(A–C)** presents the transmission dynamics of infectious diseases with herd immunity through infection simulated by the conventional SIR model, Model A and Model B. **(D,E)** presents the proportion differences between the conventional SIR model and Model A, and between the conventional SIR model and Model B. **(F)** presents the total infections generated by the conventional SIR model, Model A and Model B with varying r_0_. All data used to create the figures can be sourced at https://github.com/william81/newSIR.herd-immunity.model.script.

In simulations without vaccination, all three models depicted the transmission dynamics according to natural herd immunity consequent to the rising proportion of immunized individuals. Figure 4 presents the transmission dynamics of infectious disease with r_0_ of 3.0 simulated in conventional SIR model, Model A and Model B. Our simulations showed that all three models performed similarly at the initial phase of outbreak when both I(t) and R(t) were relatively small as compared with the total population size, N.

According to the conventional SIR model, the total R(t) or recovered population with natural immunity would reach the level predicted by the HIT at t = 86, with total infections of 86.87%, I(t) of 19.20% and S(t) of 13.13%. In due course, more people will become infected. The infection affected up to 94.05% of the population and only 5.95% of population remained susceptible (Fig. 4A and Table 3). Model A accurately projected the infectious disease to be controlled and eradicated at the level predicted by the HIT at the end of simulation (Fig. 4B and Table 3). Model B projected the infectious disease to subside at a level higher than the HIT, with total infections of 75.60% and 24.40% of the population remained susceptible at the end (Fig. 4C and Table 3).

**Table 3:** Outputs of simulations using the conventional SIR model, Model A and Model B.

Outputs	Conventional SIR model	Model A	Model B
**Natural immunity model**			
**HIT was achieved at**	t = 86	t = 195	t = 98
S(t)	13.13	33.34	26.19
I(t)	19.20	0.01	6.49
Total infections	86.87	66.66	73.81
R(t)	67.67	66.65	67.32
**At the end of simulation (t = 200)**			
S(t)	5.95	33.34	24.40
I(t)	0	0.01	0
Total infections	94.05	66.66	75.60
R(t)	94.05	66.65	75.60
**Natural and vaccine immunity model**			
**(A) ν = 1.0% population per unit t**			
**HIT was achieved at**	t = 67	t = 67	t = 67
S(t)	32.79	32.98	32.98
I(t)	0.06	0	0
Total infections	0.21	0.02	0.02
Total immunized	67.15	67.02	67.02
Through infection	0.15	0.02	0.02
Through vaccination	67.00	67.00	67.00
**At the end of simulation (t = 500)**			
S(t)	31.40	32.98	32.98
I(t)	0.01	0	0
Total infections	1.60	0.02	0.02
Total immunized	68.59	67.02	67.02
Through infection	1.59	0.02	0.02
Through vaccination	67.00	67.00	67.00
**(B) ν = 0.5% population per unit t**			
**HIT was achieved at**	t = 100	t = 131	t = 131
S(t)	27.33	33.48	33.47
I(t)	5.88	0.03	0.03
Total infections	22.67	1.02	1.03
Total immunized	66.79	66.49	66.50
Through infection	16.79	0.99	1.00
Through vaccination	50.00	65.50	65.50
**At the end of simulation (t = 300)**			
S(t)	16.61	33.47	33.46
I(t)	0	0	0
Total infections	33.39	1.03	1.04
Total immunized	83.39	66.53	66.54
Through infection	33.39	1.03	1.04
Through vaccination	50.00	65.50	65.50
**(C) ν = 0.1% population per unit t**			
**HIT was achieved at**	t = 87	t = 134	t = 107
S(t)	16.05	32.72	29.00
I(t)	18.87	0.86	4.67
Total infections	75.25	53.88	60.30
Total immunized	65.08	66.42	66.33
Through infection	56.38	53.02	55.63
Through vaccination	8.70	13.40	10.70
**At the end of simulation (t = 200)**			
S(t)	6.94	32.33	27.37
I(t)	0	0.01	0
Total infections	84.36	54.27	61.93
Total immunized	93.06	67.66	72.63
Through infection	84.36	54.26	61.93
Through vaccination	8.70	13.40	10.70

Our simulations revealed that the control of transmission of infectious disease occurred after t = 50 in both Model A and Model B as compared with the conventional SIR model (Fig. 4D,E). The sensitivity analyses confirmed that total infections generated by the conventional SIR model at the end of simulation were well above the HIT across all r_0_ values. Total infections generated by Model A at the end of simulation were accurately predicted by the HIT across all r_0_ values, while total infections generated by Model B predicted the HIT at smaller r_0_ values and started to deviate away from the HIT at higher r_0_ values (Fig. 4F).

Model A simulated the infectious disease to subside at a level predicted by the HIT accurately across all r_0_ values by allowing the transmission rate to decline after individuals became infected. However, it might be too soon to assume that the risk of contact infection and transmission rate would reduce immediately in such a transition. Model B was more in line with the fundamentals of infectious disease stages as it allowed the transmission rate to decline when immunity was developed following recovery.

### Transmission dynamics of infectious diseases with natural and vaccine-induced herd immunity

**Figure 5:**
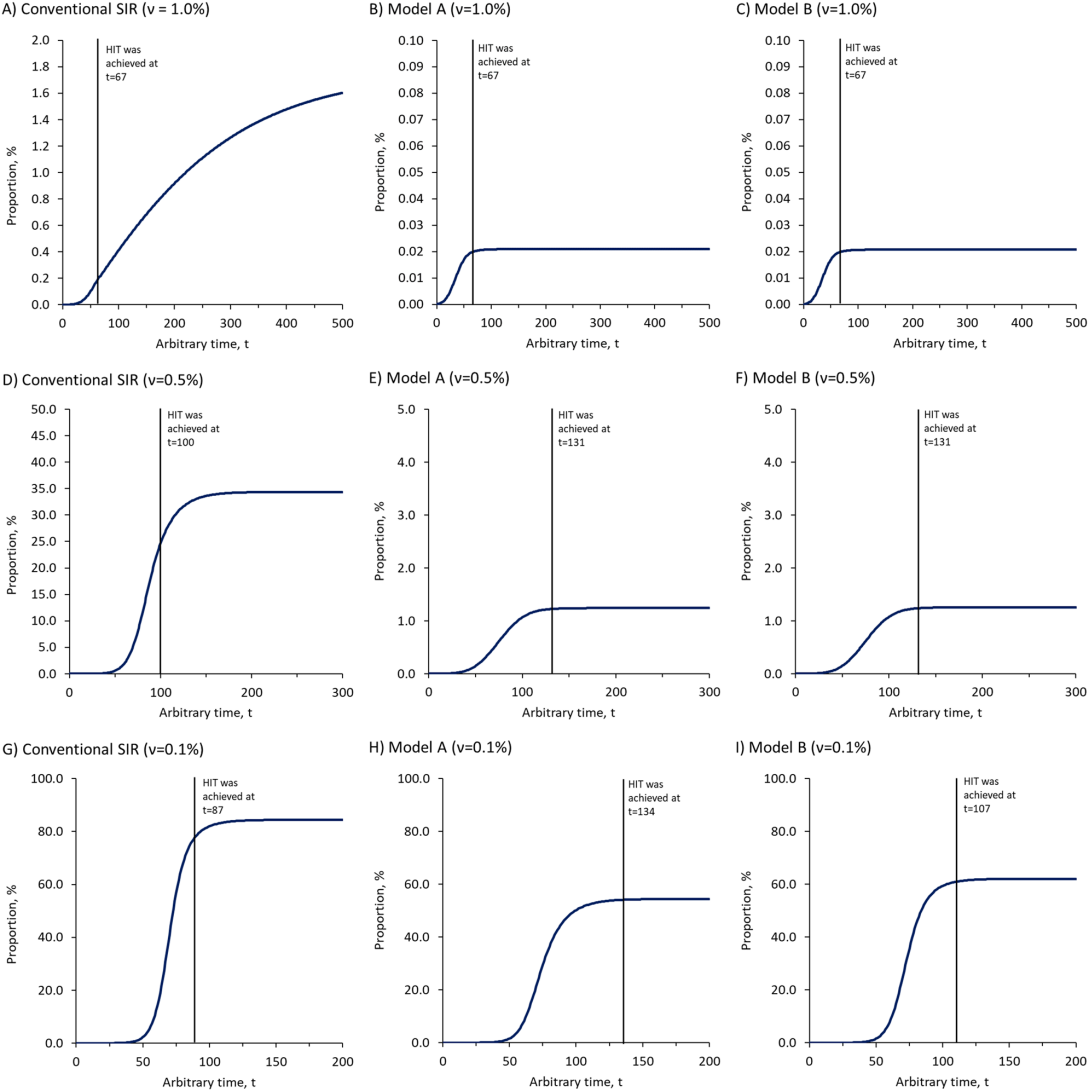
The dynamics of total infections simulated by vaccine models. **(A–C)** present the dynamics of total infections at vaccination rate, ν = 1.0% population per unit t. The HIT was reached at t = 67 in all three models. **(D–F)** present the dynamics of total infections at ν = 0.5% population per unit t. The HIT was reached at t = 100 in the conventional SIR model, and at t = 131 in both Model A and Model B. **(G–I)** present the dynamics of total infections at ν = 0.1% population per unit t. The HIT was reached at t = 87 in the conventional SIR model, at t = 134 in Model A, and t = 107 in Model B. The time to reach the HIT was marked by vertical lines. All data used to create the figures can be sourced at https://github.com/william81/newSIR.herd-immunity.model.script.

With the framework as shown in Fig. 3, all three models described the transmission dynamics of infectious diseases with vaccine-induced herd immunity at different vaccination rates. At a very high vaccination rate (ν = 1.0% population per unit t), our simulations showed that total infections would continue to increase in a steady trend even after the HIT was achieved in the conventional SIR model (Fig. 5A). The conventional SIR model failed to demonstrate either control or eradication of infectious diseases even at a high vaccination rate after the HIT was achieved, let alone lower vaccination rates. In contrast, both Model A and Model B projected the infectious disease to be controlled and eradicated when the total immunized population reached the HIT. At a very high vaccination rate, both Model A and Model B performed similarly and generated the same outcome with total infections controlled at 0.02% after the HIT was achieved at t = 67 and until the end of the simulation (Fig. 5B and C, Table 3).

At a lower vaccination rate (ν = 0.5% population per unit t), total infections continued to increase at a higher rate even after the HIT was achieved at t = 100 in the conventional SIR model (Fig. 5D, Table 3). At t = 100, the total immunized population reached 66.79% of the population, including 16.79% of the population immunized through infection and 50.00% of the population immunized through vaccination. The infectious disease appeared completely out of control and continued to infect more people in the population, causing the total infections to increase by another 10.72% and reached 33.39% by the end of simulation (t = 500). Both Model A and Model B continued to project the infectious disease to be controlled even at a lower vaccination rate. At ν = 0.5% population per unit t, the total immunized population would reach the HIT at t = 131 in both models, with total infections controlled at 1.02% to 1.03% in Model A and 1.03% to 1.04% in Model B, respectively (Fig. 5E,F, Table 3).

At the lowest vaccination rate (ν = 0.1% population per unit t), the herd immunity was largely contributed by infection or natural immunity. The total immunized population would reach the level predicted by the HIT at t = 87, with total infections of 75.25% in the conventional SIR model. Subsequently, total infections continued to increase by 9.11% and reached 84.36% at the end of simulation (Fig. 5G, Table 3). As for Model A, the total immunized population would reach the level predicted by the HIT at t = 134, with total infections of 53.88%. At the end of simulation, total infections only increased by another 0.39% to 54.27% in Model A (Fig. 5H, Table 3). In model B, the HIT was reached at t = 107, with total infections of 60.30%. At the end of simulation, total infections continued to increase only by another 1.09% in Model B (Fig. 5I, Table 3).

### Influenza disease burden in the United States from 2010 to 2019

**Figure 6:**
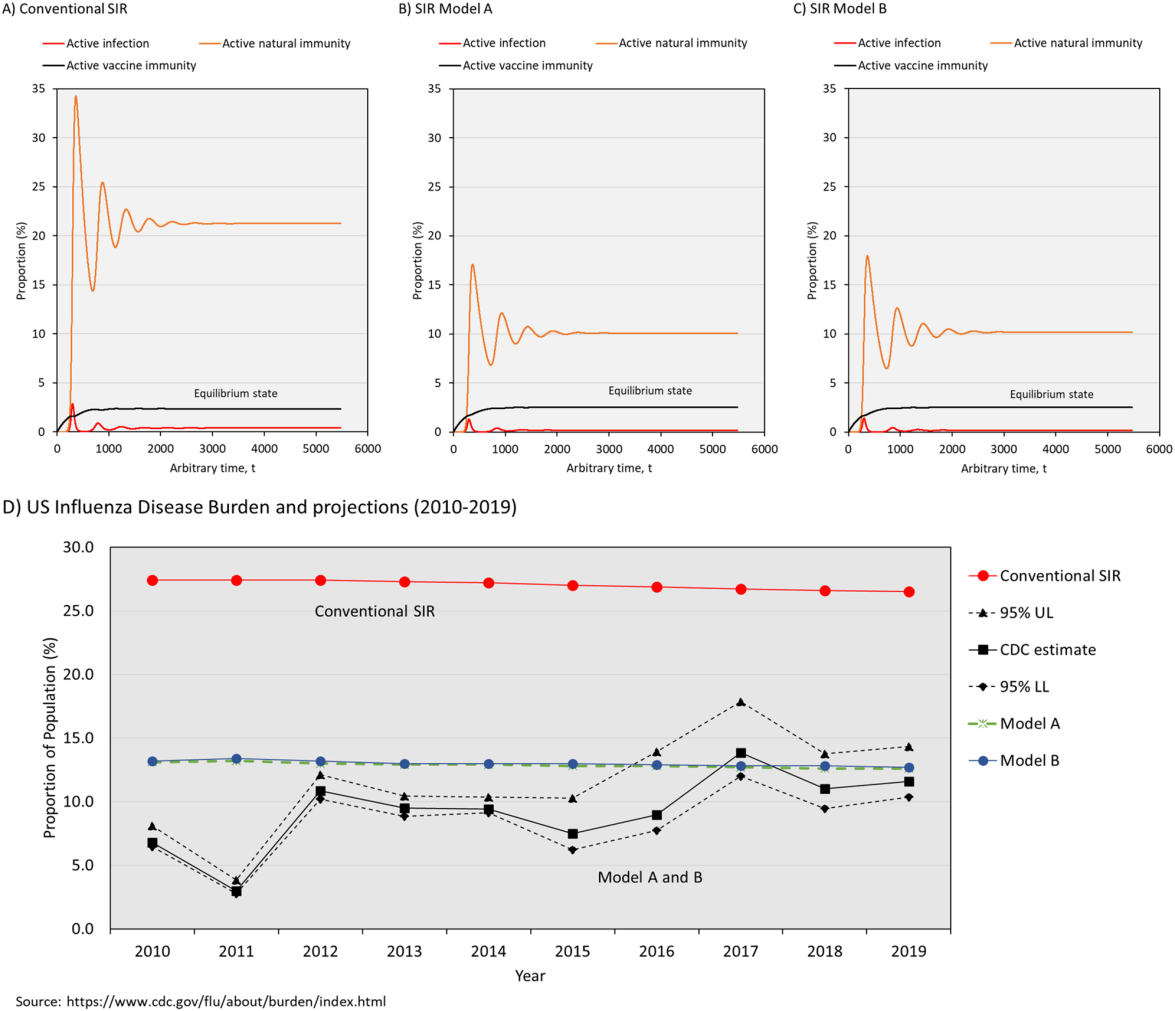
The projected disease burden of seasonal influenza in the United States from 2010 to 2019. Transmission dynamics of seasonal influenza projected by **(A)** conventional SIR model, **(B)** Model A, and **(C)** Model B. **(D)** Comparison of projected disease burden by the conventional SIR model, Model A and Model B with estimates reported by the CDC from 2010 to 2019. Model A and B provided more accurate projections than the conventional SIR model. All data used to create the figures can be sourced at https://github.com/william81/newSIR.herd-immunity.model.script.

Our simulations show that both Model A and Model B outperformed the conventional SIR model in tracking the influenza disease burden in the United States from 2010 to 2019 (Fig. 6). The CDC estimated the influenza disease burden of symptomatic illness in the United States to fluctuate between 3.0% and 13.8% from 2010 to 2019. Both Model A and Model B projected the disease burden at 12.6% to 13.4% and these values matched the CDC estimates. Due to a small r_0_ and short contagiousness duration, the difference between Model A and Model B was hardly noticeable. Despite having a higher degree of population immunity, the conventional SIR model overestimated the transmission dynamics and disease burden of seasonal influenza in the United States by approximately two to nine folds (26.5% to 27.4%).

## Discussion

The key to modelling the transmission dynamics of infectious diseases with herd immunity is to associate the transmission rate with the reduced risk of contact infection after acquiring immunity. The inclusion of the second risk component into the force of infection would not affect the early dynamics as shown in the simulations. However, such fundamental transformation can lead to very different results as presented by both Model A and Model B. Infectious diseases modelled using the conventional SIR model appear to be overly aggressive, and nearly impossible to demonstrate control or eradication even with herd immunity. This raises a crucial concern of using the conventional SIR model or its variants to simulate the late phase of the COVID-19 pandemic or other infectious diseases through natural immunity and vaccine immunity.

Model A successfully demonstrates control and eradication of infectious disease at the level predicted exactly by the HIT. This is achieved by letting the transmission rate declines along with the reduced risk of contact infection before the development of immunity. Due to the violation of the assumption of infection stages, Model A is not recommended to be used as the modelling framework for simulating the impact of herd immunity. In addition, our simulations show that the same violation might have occurred in the threshold theorem proposed by Dietz in 1975, which explains why projections of Model A are well matched by the HIT across all r_0_ values. Hence, further studies are required to investigate and re-establish the right threshold for estimating the level of herd immunity required for the control and eradication of infectious diseases.

During the course of an infection, infected individuals enter sequential stages or periods with regards to infectivity and clinical manifestations and immunity develops only after recovery or vaccination. Besides being protected from re-infection, individuals with immunity would offer indirect protection against the transmission of pathogens among susceptible individuals. Among the new SIR models, only Model B satisfies the underlying assumption of infection stages, while meeting the theoretical principle of herd immunity. Our sensitivity analyses further confirmed that Model B provides the right mechanistic framework for modelling the transmission dynamics of infectious diseases with regards to both natural and vaccine-induced immunity. In the influenza simulation, we prove that the newly proposed modeling framework provides a more accurate projection of disease burden than the conventional SIR model. In addition, our sensitivity analyses reveal that natural herd immunity might not be effective for infectious diseases with very high reproduction numbers. Furthermore, Model B can be modified to account for the impact of duration required for developing complete immunity in scenarios where immunity is fully developed after a period following recovery or vaccination.

Importantly, the new models show that the transmission rate may decline rapidly after a particular time, depending on the population size, contact rate and duration of infection. This might explain the immediate fall of COVID-19 cases in some countries like the United States, United Kingdom and Indonesia, shortly after the rapid rollout of the mass vaccination campaign against the COVID-19 pandemic prior to achieving the herd immunity threshold. Moreover, the newly developed model may provide a better framework for explaining the steady fall of COVID-19 cases in India even without vaccination since September 2020^33^. Many researchers attributed the fall of COVID-19 cases without vaccination in India to natural immunity and younger population demographic. A national serological survey conducted by the Indian Council of Medical Research (ICMR) revealed that up to 21% or 290 million of the adult population in India had developed immunity against the COVID-19 virus^34^.

Currently, more than 230 million individuals have been infected by the novel coronavirus with a death toll surpassing 4.7 million^33^. At the same time, many countries have started mass vaccination with the hope to end the COVID-19 pandemic with vaccine-induced herd immunity. Therefore, the use of the right modelling framework for herd immunity becomes critically important and relevant to support post-vaccination public health planning and preparedness against the pandemic.

## Conclusion

Our new models successfully demonstrate control and eradication of pandemic with herd immunity, but at the same time shows that natural herd immunity might not be sufficiently effective in infectious diseases with high reproduction numbers. We think the key to simulating the transmission dynamics of infectious diseases with herd immunity is to associate the transmission rate with the reduced risk of contact infection following recovery or vaccination as outlined in Model B. This can be attained by incorporating the inverse proportion of immunized individuals into the force of infection in the SIR model. Further studies are required to establish the right threshold for herd immunity based on the newly proposed theory and framework for the transmission dynamics of infectious diseases in a randomly mixed population.
